# Status and determinants of small farming households' food security and role of market access in enhancing food security in rural Pakistan

**DOI:** 10.1371/journal.pone.0185466

**Published:** 2017-10-27

**Authors:** Umar Ijaz Ahmed, Liu Ying, Muhammad Khalid Bashir, Muhammad Abid, Farhad Zulfiqar

**Affiliations:** 1 Department of Agricultural and Resource Economics, Faculty of Social Sciences and Humanities, Muhammad Nawaz Shareef University of Agriculture, Multan, Pakistan; 2 College of Economics and Management, Huazhong Agricultural University, Hubei, Wuhan, P.R. China; 3 Hubei Collaborative Innovation Centre for Grain Industry, Yangtze University, Hubei, Jingzhou, P.R. China; 4 Institute of Agricultural and Resource Economics, University of Agriculture Faisalabad, Faisalabad, Pakistan; 5 Agriculture Policy, USPCAS-AFS, University of Agriculture, Faisalabad. Faisalabad, Pakistan; 6 Centre for Climate Research and Development, COMSATS Institute of Information Technology, Tarlai Kalan, Islamabad, Pakistan; 7 Department of Economics, COMSATS Institute of Information Technology, Tarlai Kalan, Islamabad, Pakistan; Universidade de Brasilia, BRAZIL

## Abstract

In most of the developing countries, lack of resources and little market accessibility are among the major factors that affect small farming household food security. This study aims to investigate the status of small farming households’ food security, and its determinants including the role of market accessibility factors in enhancing food security at household level. In addition, this study also determines the households’ perception about different kinds of livelihoods risks. This study is based on a household survey of 576 households conducted through face-to-face interviews using structured interviews in Punjab, Pakistan. Food security status is calculated using dietary intake method. The study findings show that one-fourth of the households are food insecure. The study findings reveal that farm households perceive increase in food prices, crop diseases, lack of irrigation water and increase in health expenses as major livelihood risks. Further, the results of logistic regression show that family size, monthly income, food prices, health expenses and debt are main factors influencing the food security status of rural households. Furthermore, the market accessibility factors (road distance and transportation cost) do significantly affect the small farming household food security. The results suggest that local food security can be enhanced by creating off-farm employment opportunities, improved transportation facilities and road infrastructure.

## Introduction

Food security is a complex phenomenon and may be seen as an integration of three core dimensions i.e. food availability, accessibility and utilization [1] The problem of food insecurity is not only caused by insufficient supply of food, but also due to the lack of purchasing power and access at national and household levels. Therefore, despite gains in global food production and food security over the last three decades [2], still more than 800 million people are undernourished and almost all of them belong to the developing countries [3]. Furthermore, growing population coupled with increased intensity of environmental extreme events i.e. floods, droughts, extreme variability in temperature and rainfall has increased the pressure on current food production systems and has threatened the current food security in most of the developing countries [4]. Due to higher food demands and reduced crop productivity, the higher food prices may further negatively affect the food access and availability for low income and already poor households.

Like many other developing countries, Pakistan is considered to be one of the countries most affected by food insecurity, poverty and environmental disasters [5]. About two-third of the population in Pakistan lives in rural areas and directly or indirectly relies on the agricultural sector for their food and livelihood [1,6]. However, the majority of the rural population consists of small-scale households owning less than 2 hectares of land and with little access to resources and services [7]. Therefore, besides having self-sufficiency in overall crop production at national level, the small-scale rural communities in Pakistan are still facing the problems of reduced crop productivity, food insecurity and poverty [8]. In addition, low adaptive capacity to manage environmental disasters is also adversely affecting the agricultural productivity and local food security in Pakistan [7].

The food security gains are not transferred at local level due to complex food supply chain system and prevailing risks and vulnerabilities along the supply chain. The absence of proper food transport infrastructure and little access to food and market are some of the many factors that can affect food insecurity in the Asian region [9–10]. Food security situation at local level may be improved by focusing on access to food and food distribution systems [11–12]. Better food distribution system mainly depends on food and market access. Accessibility refers to ‘ease to use’ or ‘openness to’ to a certain facility [12].

Market accessibility could be considered as one of the most important factors affecting rural food security. It is often linked with various other stakeholders e.g. processors, traders, and retailers [13]. Being the producers and consumers at the same time, market access plays two-way function for rural households. On one hand they use the market to buy inputs or to sell farm produce while on the other hand, they use it to buy food and non-food items in order to sustain their living standards [14]. Market access may be hindered due to long distances from farm to market, transportation cost and market information [15]. Hence, better infrastructure and easy market access can play an important role in sustaining local food security through reduced transportation cost and food prices [16]. Market access could be defined in various ways like using proxies such as travel time, distance and cost [17]. In addition to market access, access to other institutional services such as extension and credit are also important to enhance local access and utilization of food [12].

To date, a few studies [17–26] have been conducted focusing on the different dimensions of rural food security in Pakistan. However, little focus is given to the role of market accessibility in enhancing access to food and related food security of rural households. Therefore, keeping in view the existing research gaps, this study aims to investigate the impact of market accessibility on rural household food security along with causes of food insecurity. Specifically, this study has three objectives: 1) to measure rural household food security status and indicators in rural areas of the Punjab province, Pakistan; 2) to identify the key causes of food insecurity among rural households; 3) to determine the role of market accessibility along with other socio-economic factors in enhancing rural household food security.

## Methodology

### Study area description

The study was mainly conducted in Punjab province, which is the most populous province in Pakistan. Punjab was selected as main study area due to its importance in the national economy and its share in national agricultural GDP (51 percent) and cereal production [27–28]. Geographically, Punjab province is located at 30°00' N, 70°00' E in semiarid lowland zone [29]. It is a fertile agriculture region having a large irrigation system and contributing well to the development of economy [30]. The mean annual minimum and maximum temperature in Punjab ranged from 16.3 to 31.9°C over the period 1970–2001. Following [31], Punjab province could be divided into five agro-climatic zones: wheat-rice zone, wheat-cotton zone, mixed zone, *barani* (arid) zone and low-intensity zone.

### Sampling and data collection

The study used a multi-stage stratified sampling technique to select study sites and 576 farm households from three agro-climatic zones. In the first stage, to select study districts we divided province into five strata based on agro-climatic zones (Table 1). In the second stage, we selected twelve districts out of thirty-six districts using stratified purposive sampling technique. The strata were not identical in terms of district numbers, a proportionate sample was drawn from each stratum using the formula [32]:

$$n_{i}=n\;(\frac{N_{i}}{N})$$ (1)

Where:

i = 1–5 strata
n_i_ = No. of districts in ith stratum
n = Total number of selected districts (12)
N_i_ = Total number of districts in ith stratum
N = Total number of districts (36)

**Table 1:** Agro-climatic zones of Punjab and study districts*.

	Agro-climatic Zones of Punjab Province				
	Zone 1: Wheat-Rice Zone	Zone 2: Wheat-Cotton Zone	Zone 3: Mixed Zone	Zone 4: *Barani* (Arid) Zone	Zone 5: Low Intensity Zone
Districts	Sialkot	Bahawalpur	Sargodha	Attock	D.G. Khan
	Gujrat	Bahawal Nagar	Khushab	Chakwal	Rajan Pur
	Gujranwala	Multan	Jhang	Jhelum	Muzzafar Garh
	Mandi Bahaudin	Sahiwal	Faisalabad	Rawalpindi	Layyah
	Sheikhupura	Rahim Khan	Okara		Mianwali
	Lahore	Khanewal	Toba Tek Singh		Bhakkar
	Kasur	Vehari	Chiniot		
	Nankana Sahib	Pakpatan			
	Narowal	Lodharan			
	Hafizabad				

Source: Pinckney (1989)

* The highlighted districts are the districts where the study was actually carried out.

According to the selection criteria, three districts were selected from wheat-rice, wheat-cotton and mixed zones, one from the *barani* (arid) zone and two from low-intensity zones. The districts were selected keeping in view the homogeneity in the yields of five major crops (wheat, rice, sugarcane, cotton and maize). Table 1 shows the twelve selected districts (highlighted). In the third stage, four villages were randomly selected from each district. In the fourth stage, we randomly selected 12 households from each village. These criteria were used to form the sample of 576 households [15].

A pre-tested structured questionnaire (S1 Table) was used to collect various kinds of information from sample farm households, i.e. socio-economic characteristics, cropping techniques, input use, outputs and households’ access to market, credit and extension services. The household survey was conducted keeping in view the requirements of human ethics. For this purpose, the whole data collection procedure and framework was reviewed and approved by the Proposal Defense Committee of College of Economics and Management, Huazhong Agricultural University, Wuhan, China, headed by the Dean of the college.

For this study, we conducted interviews only with male household heads because local customs do not allow female household heads to interact with male enumerators for face-to-face interviews. A verbal consent from the respondents was taken prior to starting formal interviews. Respondents were free to participate or not to the study and to opt out at any time. At the first stage, participants were told about the objectives and purpose of the study and potential outcomes of the study and further they were assured that their information will only be used for educational purposes. In second stage, a confirmation or willingness was taken from the respondent prior to the starting a formal interview. At this stage, several respondents choose not to participate in the survey and were replaced with other respondents to complete the sample size. The written consent was not obtained due to the illiteracy and hesitation of the respondents giving any written statement. We only kept the record of the participants who were willing and participated in the study. The whole consent procedure was also approved and confirmed by the committee.

### Analytical framework

#### Determining food security status

There are number of different ways to assess food security [33]. Different studies e.g., [20, 22, 34–41] used different methods to measure food security. Among various methods, the calorie intakes method is one of the most popular methods to measure the extent of food security, which is used in this study [25, 42]. In next step, to measure the rural household food security status and indicators, we employed Dietary Intake Assessment (DIA). Because our targeted households have these characteristics i.e. 1) they are small farmers and belong to lowest income group; 2) they consider filling stomachs to maintain a subsistence level of living instead of choosing the food with the nutritional or taste values and 3) lastly, they are most vulnerable ones to be food insecure [1]. Dietary Intake Assessment (DIA) or Calorie Intake Method is a recall method for usually 7 days. This is a widely-used method for food security measurement. The estimates largely depend on the memory of the respondent and weighted values used for food before and after consumption. This method has some better and unique features than that of FAO and HIES methods [1]. The advantage of this method is that it measures the food consumed directly. But the cost of this method is very high and it needs highly trained and experiences researchers to collect and enter data into spreadsheets [41].

For this purpose, we collected data on per capita calorie intake of farm household during the last seven days prior to the interview day [25]. Here it is important to mention that the household information on calorie intake was asked from an individual household head, not all the members of the household. We did not ask the dietary consumption data of other household members from the respondent household heads because in most of the cases they were unaware of what other members of household had consumed during last seven days. Given the limited time and scope of the study, it was not possible to interview every member of 576 households in the study regions to collect their dietary intake data. Instead we preferred to use Adult Consumption Equivalents to calculate dietary intake of household members of different age and gender [43]. This method is widely used by many studies conducted in different South Asian countries [43]. This method used dietary consumption data to compute the average daily consumption of various groups based on age, gender and type of work. They expressed these intakes as per consumption unit (CU) per day. One consumption unit represents calorie consumption of an average adult man, weighing 60 kg, doing sedentary type of work. While the other coefficients are calculated based on calorie requirement proportionately. We assigned a consumption unit value one to the household head and calculated the proportional dietary intake of other members with different age, gender and type of work proportion based on consumption unit given in (S2 Table). For example, a household consists of 3 members including himself, his wife (adult female; moderate) and a child between 5–7 years old. Suppose the household head has consumed on average 2000 kcal per day, then according to the (S2 Table), the daily caloric intake of his wife will be 1800 (0.9x2000) kcal and of his child will be 1200 kcal (0.6x2000). This implies that average per capita daily caloric intake would be 1666 kcal. After calculating the per capita household’s caloric intake, it was compared with per capita threshold for food security (2450 Kcal/day) [44]. Following this criterion, a household was considered food secure if respondents used to maintain this daily caloric threshold and was assigned value “1” and zero otherwise. The formula to measure the household food security status can be written as:

$${RHFS}_{i}=\sum_{i=1}^{n}{FS}_{i}-Th\geq 0$$ (2)

Where RHFS is the rural household food security for *i*th household which takes value “1” if farm household is food secure and zero if farm household is food insecure and Th stands for the threshold level (per capita 2450 Kcal/day).

The other food security measures as presented by various studies (e.g. [45, 46, 47 & 48]) are as follows;

$$p=\frac{1}{M}\sum_{i=1}^{m}G_{i}$$ (3)

$$\mathrm{and}\;G_{i}={(FS}_{i}-Th)/Th$$

Where *p* shows the shortfall/surplus index, *G*_*i*_ is the deficiency or surplus faced by *i*th household, *FS*_*i*_ is the average daily calorie available to the *i*th household, m is the number of households that are food secure (food surplus index) or food insecure (for shortfall index).

#### Measuring food insecurity gaps

Food insecurity gap measured the extent at aggregate level to which households are below (or above) the food security line. In implementing food security policies and programs, the values of the index could be monitored over time and compared among different groups of the population.

The Total Food Insecurity Gap may be calculated as [48];

$$TFIG=\frac{1}{M}\sum_{i=1}^{m}\frac{\left(Th-{FS}_{i}\right)}{Th}$$ (4)

Where, TFIG is the total food insecurity gap, which indicates the depth of food insecurity among the food insecure farming households and M is the number of food insecure farming households. The squared Food Insecurity Gap, which indicates the severity of food insecurity among households may be given as [48];

$$SFIG=\frac{1}{M}\sum_{i=1}^{m}{\left(\frac{\left(Th-{FS}_{i}\right)}{Th}\right)}^{2}$$ (5)

#### Determining impact of market accessibility on food access: logistic regression

In order to determine the impact of market accessibility and other socio-economic factors, we used binary logistic regression model. The general logistic model may be written as;

$$logit(\delta_{i})=\beta_{0}+\beta_{i}X_{i}+\omega_{i}$$ (6)

Where; *β*_*0*_ is the intercept, *X*_*i*_ is the vector of explanatory variables used in the model (see Table 2) and β_i_ shows the estimated coefficients of the explanatory variables. *ω*_*i*_ is the error term.

**Table 2:** Description of variables used in the binary logistic regression model.

Variables	Description	Variable type
**Dependent Variable**		
Food security status	Food Security status of the household. It takes value 1 if household is food secure and zero otherwise	Binary
Independent Variables		
Age	Age of household head in years	Continuous
Education	Education level of the household head	Continuous
HH size	Total members in the household	Continuous
Earning members	Total earning hands in household	Continuous
Monthly income	Monthly income of the households	Continuous
Monthly food expenses	Monthly food expenses of the households	Continuous
Distance to road	Distance to paved road in kilometers	Continuous
Distance to market	Distance to output market in kilometers	Continuous
Transportation cost	Transportation cost to output market	Continuous
Employment loss	Risk to livelihood loss. It takes value 1 if yes and zero otherwise	Binary
Health expenses	Risk to livelihood loss. It takes value 1 if yes and zero otherwise	Binary
Food prices	Risk to livelihood loss. It takes value 1 if yes and zero otherwise	Binary
Debt	Risk to livelihood loss. It takes value 1 if yes and zero otherwise	Binary
Crop diseases	Risk to livelihood loss. It takes value 1 if yes and zero otherwise	Binary
Irrigation water	Risk to livelihood loss. It takes value 1 if yes and zero otherwise	Binary
Bad climate	Risk to livelihood loss. It takes value 1 if yes and zero otherwise	Binary

Table 2 shows the description and types of explanatory variables used in the study. Study uses both continuous and binary variables.

Model Prediction Success (MPS), Hosmer and Lemeshow (H-L) and pseudo R^2^ [49–51] were measured to describe the overall goodness of fit of the binary logistic model. The estimated coefficients *β*_*i*_ only give the direction of impact of the independent variable on the binary dependent variable [50] and do not explain to what extent the probability of the *i*th household to be food secure will be changed with the change in the value of explanatory variable. Therefore, to better interpret the results, we calculated the marginal effects as suggested by Abid et. al. [28], where marginal effect describes the magnitude of the effect of a unit change in the explanatory variable on the probability of a household being food secure.

## Results

### Descriptive statistics

The descriptive statistics of the sociodemographic characteristics of the participants are presented in Table 3. The average age of the household head was about 47 years. A household was consisting of on average 7 members along with 2 earning members. The Mean distance to the paved road and output market was about 3 and 14 kilometers respectively. mean transportation cost per acre to output markets was about US$ 46. The average per capita calorie intake was about 3175 kcals per day. On average, each farm household earned monthly US$ 238.

**Table 3:** Descriptive statistics of continuous variables.

Variables	Unit	Minimum	Maximum	Mean (Sd)
Total Household Members	No’s	2	26	6.98 (2.8)
Total Earning Hands	No’s	1	5	1.6 (0.9)
Households’ Head Age	Years	24.0	80.0	47 (9.8)
Distance to Paved Road	Km	0.0	18	2.8 (3.3)
Distance to Output Market	Km	0.0	30	13.9 (6.9)
Transportation Cost to output markets	US$	0.0	643.2	45.9 (59.5)
Per Capita Calorie Intake per day	Kcal	1218.8	9638.6	3175.4 (1171)
Monthly Income	US$	76.9	446.8	(238.4) 101
Total Number of Participants (N)				576

### Small farm households’ food security status

Calorie intake method was used to calculate the food security status of the small farming households of the study area. Here it is important to mention that to calculate per capita household caloric intake, we did not directly collect information on dietary intake of their household members. Instead we used Adult Consumption Equivalents to calculate the average dietary intake of each household member of different age and gender which is the proportion to the dietary intake of the household head. Results of the study presented in Table 4 show that more than three-fourth of the farming households were found to be food secure (78%) as their per capita daily caloric intake was equal or beyond the threshold (2450 kcal/day). While the one-fourth of the households (22%) were found to be food insecure. Surplus and shortfall indices are 0.21 and 0.48. Total food insecurity gap for all households and per household is 0.2092 and 0.047. Squared food insecurity gap is calculated as 0.060.

**Table 4:** Food security indicators.

Indicators	Value
Total Number of Participants	576
Food Secure Households	447 (77.6%)
Food Insecure Households	129 (22.4%)
Surplus Index	0.21
Shortfall Index	0.48
Total Food Insecurity Gap (TFIG) for all households	0.2092
Total Food Insecurity Gap (TFIG) per households	0.047
Squared Food Insecurity GAP (SFIG)	0.060

### Participants’ perceptions of livelihood risks

**Fig 1:**
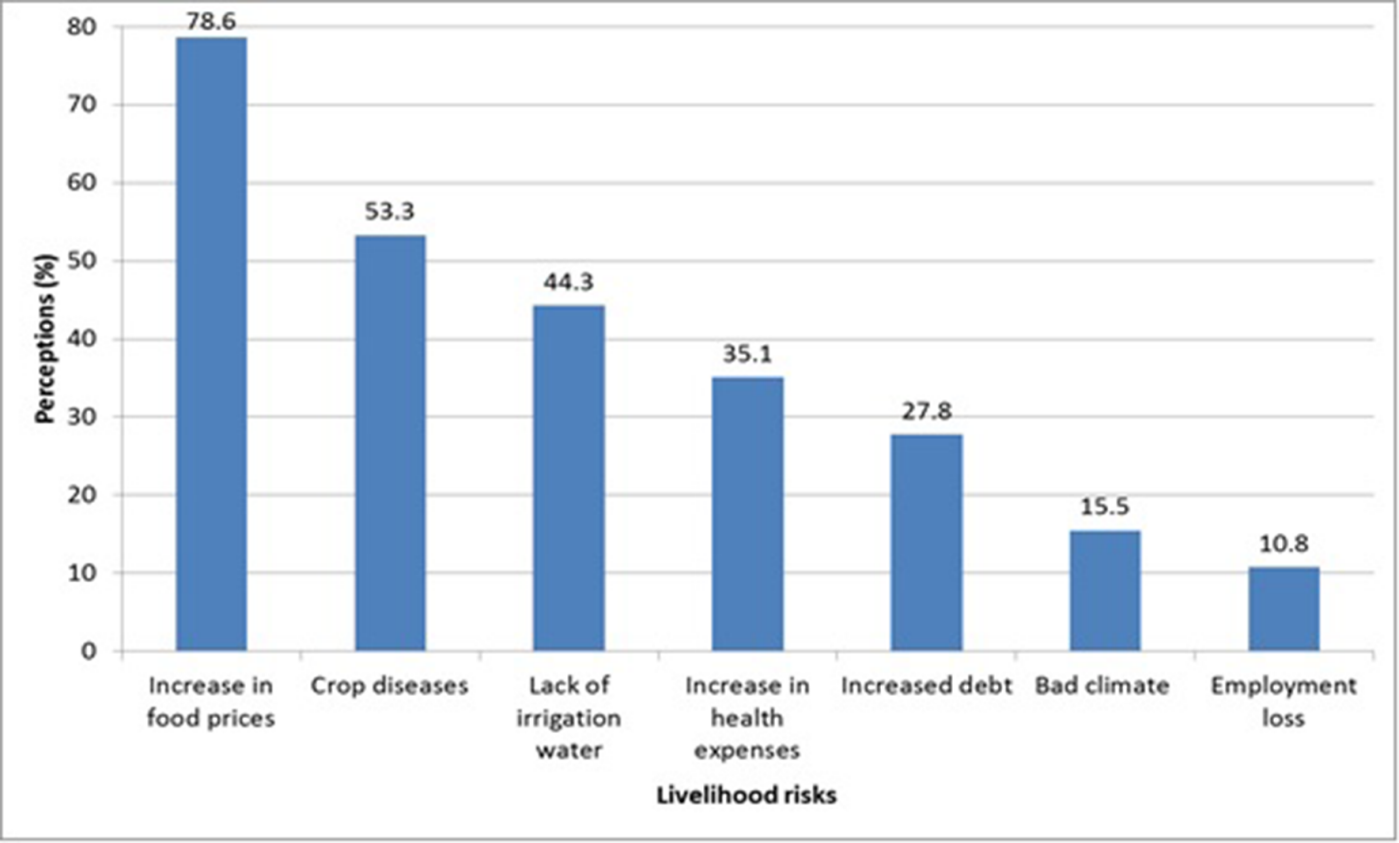
Participants’ perception of livelihood risks.

Further, we asked farmers to rank their perceptions about different kind of risks that may affect their livelihoods. According to the study findings as shown in Fig 1, farmers perceived increase in food prices (79%), crop diseases (53%), lack of irrigation water (44%) and increase in health expenses (35%) as major livelihood risks. Other livelihood risks reported by farmers include increase in debt, loss of employment and bad climate.

In next step, we used binary logistic regression analysis to determine the factors affecting small household food security in the study area using logistic regression analysis. The study findings presented in Table 5 shows that the model has a good fit (82 percent) and non-significant Hosmer and Lemeshow.

**Table 5:** Determinants of small farmer’s household food security.

Variables	β	*Standard Error*	*coefficients*	*Marginal Effects*
Constant	3.714385***	0.789	41.03335	0.08627
Age	-0.016964	0.014	0.98318	-0.00424
Education	0.078623	0.088	1.08180	0.01963
HH size	-0.395357***	0.060	0.67344	-0.09508
Earning members	0.288140	0.178	1.33394	0.07056
Monthly income	0.000032***	0.000	1.00003	0.00001
Monthly food expenses	-0.000004	0.000	1.00000	0.00000
Distance to road	-0.083730**	0.035	0.91968	-0.02090
Distance to market	0.015687	0.018	1.01581	0.00392
Transportation cost	-0.000103***	0.000	1.00010	-0.00003
Employment loss	0.570127	0.392	1.76849	0.13155
Health expenses	-0.470874*	0.244	0.62446	-0.11143
Food prices	-0.530262*	0.306	0.58845	-0.12367
Debt	-0.476762*	0.264	0.62079	-0.11267
Crop diseases	-0.082140	0.248	0.92114	-0.02050
Irrigation water	0.030496	0.248	1.03097	0.00762
Bad climate	-0.117778	0.321	0.88889	-0.02934
Total No. of respondents	576			
Total No. of Independent Variables	16			
Model Prediction Success	82.10%			
Log-likelihood ratio	466.5			
Cox & Snell R^2^	0.223			
Nagelkerke R^2^	0.341			

*, **, *** are significant at 1%, 5% and 10% level of significant

The study results show that small household food security has a significant and positive relationship with monthly income and negatively related with household size, distance to road, transportation cost, employment loss, health expenses, risks of increase in food prices and debt (Table 5).

## Discussion

Study findings show that about three-fourth of the households are food secure and about one-fourth are food insecure. However, the surplus and shortfall indices tell us real story. According to the both indices, about 21% of the households fall below the security line and about 48% are fully food secure, while a reasonable number of farm households (31%) unfortunately are at the edge of food security line, falling neither below nor above the reference line. This implies that due to high dependence on climate-sensitive agriculture and low access to adaptive resources, there are chances that many farm households may become food insecure in near future. On the other hand, only 48% of the farm households were found food secure. Similar trends are observed in some parts of Punjab, Pakistan [17] and other developing countries with similar conditions such as Nigeria [52–54]. Further, the food insecurity gap shows that food insecure households were consuming 5% per capita less calories than food secure household. In line with other studies [55–56], the problem of food insecurity was 6% severe in the study area. However, the method used to calculate per capita average daily caloric intake may have some limitations as it does not directly capture the dietary intake of different age and gender groups among households and totally rely on household head’s consumption pattern. Therefore, the results generated by may be overstated or understated due to several reasons. For example, some of the studies found that Adult Consumption Equivalents calculations were 20% above or below than actual dietary intake [43]. However, these results may be improved through carefully constructing the questionnaire to capture background information and caloric intake of respondent household head.

About the perceptions of livelihood risks, farmers mainly perceived increase in food prices, crop diseases and lack of water as major risks to their livelihoods. These risks are very valid given the imperfect market conditions and changing environmental conditions in Pakistan and its impacts on local agricultural communities. According to an estimate, small income households spent nearly 70 percent of their income on food items. Hence, any increase in food prices will affect farmers purchasing power and may disturb their balance sheet and may also affect their food security status and the use of other utilities. Further, this increase in food prices may also affect their spending on other items and may also alter their crop management decisions of crop choices and use of input mix. In long-run, it may affect farmers’ agricultural income and overall wellbeing. [57] also found a direct correlation between food prices and food insecurity.

Crop disease is another limiting factor that may directly affect households’ wellbeing by reducing crop productivity and food production. Effective disease control requires a lot of financial resources and technical skills that are always limited due to small-scale and unskilled farming in Pakistan. Studies also show that there is increase in the crop pest and disease attack over the years in Pakistan, which may be indirectly associated to climate change and related extreme events such as floods and droughts [4]. Since the crop disease directly affect the crop growth and limit the crop productivity, therefore this will ultimately affect farm income and household purchasing power.

Further, lack of water is another important risk perceived by farmers in the study area. This is the fact that Pakistan is one of the water scarce countries in the world and current per capita water availability is significantly declining due to over exploitation of groundwater and declining surface water. Further, changes in climate through increase in temperature and changes in rainfall distribution affect the evapotranspiration and water availability for agriculture sector in Pakistan. Shortage of water may have serious implications for crop production and may affect livelihoods and food balance of farm households who are totally dependent on agriculture for their subsistence [4]. Moreover, increase in health expenses, debt, bad climate and loss of off-farm are some other risks reported by farmers that may affect their livelihood in various ways. In other way around all mentioned risks affect farmer purchasing power due to limited income and resource and affect their food security status.

Further, the results of the logistic regression show the various important factors that determine the household’s food security status. Particularly, an increase in the household size by one member decreases the probability of a household being food secure by 10%. This implies that increase in the household size put more burden on earning members and may affect their food security status due to availability of limited resources. These results are in line with the findings of other studies [e.g. 17, 58], which also found a negative association between increase in household size and food security status. Market plays a very important role in determining the food security status of a household as it indicates the access and availability to food. Here market access is considered through three indicators such as transportation cost, access to road and market. The first indicator, distance to market is positively but insignificantly associated with food security status. However, second indicator, distance to paved road is significantly associated with food security status and shows that a one unit increase in the distance to paved road decreases the chances of a household being food secure by 2%. This implies that late access to paved road may affect farmers’ income through increase in cost of production, which may be associated with use of more labor and resources to access to paved road for marketing and selling of produce. Therefore, an increase in cost of production may lead to less farm income and may indirectly affect farm household food security status. The third indicator, the transportation cost is also negatively associated with food security status. Here, the coefficient value is very small due to lower currency unit; hence we used an incremental unit of 100 US$ to explain our results. For instance, an increase of transportation cost by US$ 100 leads to a 0.3% decrease in the probability of a household being food secure, which implies that there will be less income at the end which will also affect spending on food consumption and hence farm households may be food insecure due to this change in spending due to increased transportation cost. A couple of studies [e.g. 55–56] also revealed that increase in the cost has the negative impact on household food security status.

Contrary to the transportation cost, the monthly income does have a positive and significant impact on food security status as an increase of US$ 100 in monthly income increases the chances of a household to be food secure by 0.1%. Health expenses, food prices, and debt also have a significant impact on small farming household food security. Increases in these expenses reduce the probability of a household to be food secure by 10–12%. This implies that poor health and sanitation conditions may adversely affect household food security status due to switching expenditures from food to non-food items. These results are in accordance with a study conducted in similar conditions [58]. Similarly, higher food prices may reduce the household purchasing power and may adversely affect food security status. Furthermore, increasing debt is another limiting factor to the food security status of a household. The high transaction cost and interest rate may limit the effectiveness of credit and may have adverse impact on overall food security status. Similar to our findings, [59] also found that access to credit has a negative impact on household food security in the study area.

Here, it is important to mention that this study do not consider gender difference, that are indeed important, in the calculation of food security at farm household level due to local customs that do not allow male enumerators to interview female household heads. Hence, due to these reasons, this study only provides the male perspective of farm household heads. However, these limitations may be addressed in future by hiring female enumerators to conduct interviews of female household heads to get an equal picture of the situation based on both male and female perspectives. Other limitation of the study are the limitations of using the calorie intake as a measure of food security and the use of male household head calorie intake as a proxy to calculate calorie intake for other household members using adult equivalent unit. Another limitation of the study is the use of cross-section data for food security measure, which may be covered by conducting a panel survey up to two to three years to get reliable measures of food security status of farm household over the years.

## Conclusion

Using a household survey of 576 farmers from different agro-climatic zones of Punjab, Pakistan, this study evaluates the small farming households’ food security status and its determinants including role of market accessibility factors in defining food security at rural household level. The study findings show that the food security situation of farm household is not convincing as still a large fraction of farm households are either food insecure or fall at the edge of food security line. Further, increase in food prices, crop diseases and lack of water are major risks perceived by farmers that may affect their livelihood and food security status. These most important risks are directly or indirectly associated with current changes in climate and suggest the need for proper action to protect livelihoods of small farming communities, which are totally dependent on agriculture sector. Adaptation of the current farming systems and livelihoods to these risks could be one of the options to cope with current and upcoming problems. However, for this purpose, efforts are required both at local as well as at policy level. Further, family size, monthly income, increased food prices, debt, health expenses and more importantly market-related factors like distance to paved road and transportation cost significantly influence the household’s food security status. All this implies that more investment and focus need to be given on food distribution system and infrastructure. Easy access to market and improvement in the infrastructure will not only reduce the transportation cost but it will also improve the availability of cheap food products at local level. Further, it will also increase household purchasing power and will improve food security status at local level. In this regard, local governments also need to prioritize the provision of basic health facilities in rural areas in order to reduce the health expenses of low-income groups that ultimately may have positive impact on food security. In addition, off-farm employment opportunities need to be generated in rural areas to accommodate surplus labor from agriculture sector in order to enhance labor productivity in agriculture sector and farm income.

## Supporting information

S1 Table Questionnaire. (PDF)

S2 Table Dietary consumption units according to age and sex. (PDF)

S3 Table Food composition table for Pakistan. (PDF)
