# Supplementary material for: Status and determinants of small farming households' food security and role of market access in enhancing food security in rural Pakistan
Source: PLoS One. 2017 Oct 27;12(10):e0185466. doi: 10.1371/journal.pone.0185466 (PMC5659641; doi:10.1371/journal.pone.0185466)
Supplement: S1 Table — (PDF) [file pone.0185466.s001.pdf]

S1 Table

HOUSEHOLD FOOD SECURITY QUESTIONNAIRE

HH_ID: ____________________ Enumerator's Name: _________________________

District: ____________________ Village: ______________ Date: _____________

A.1. <u>Personal and demographic information of the households</u>

Name of the Respondent: ________________________________ Gender: Male Female

Respondent is the household head? 1) Yes 2) No,

if No, Relationship with household head __________________

Age of the Respondent: _____________ Age of the household head: ______________

Household Members: ____________________ Total earning hands: ______________

Age in Years	Male Member	Females Members	Total
< 1			
1-9			
10-17			
Adults			

Education level of household head (tick one of the followings)

1- Illiterate
2- 5 Years of schooling (Primary)
3- 8 Years of schooling (Middle)
4- 10 years of schooling (High)
5- 12 years of schooling (FA/FSc)
6- Above 12 years of schooling (Graduation)
6- Masters
7- Others (Diploma, etc.)

Decision making regarding food purchase / consumption: Household head (Male) ____ Spouse (Female) ___

Tenancy Status

1- Owner
   - Owned area (acres): ___________________
   - Rented in area (acres): _________________
   - Rented out area (acres): ________________
2- Tenant
   - Rented in area (acres): _________________

Does the household support anyone living outside the home with money or food during last 12 months?

Yes No Support value in money = RS. _______

If yes then tick all those that apply

1) Other family member(s) living in rural area
2) Other family member(s) living in urban area
3) Other family member(s) living abroad
4) Others (Specify)

Is the household getting any support (cash, food, non-food essential services & goods, etc.) from anyone outside the household at present? If yes then what are those?

Cash for food purchases **Yes** **No**

Cash for other non- food essential (school, fees, rentals, other bills) **Yes** **No**

Food (in kind) **Yes** **No**

Other (specify)

Assets at household home and farm

Sr. No.	Assets	Number	Value (Rs)
1	House		
	Car		
	Motor cycle		
	Tractor		
	TV		
	Oven		
	Refrigerator		
	Air Conditioner		
	Other Farm Equipments		

What is the source of drinking water: Ground Water Canal Water

What's your opinion about the quality of drinking water you used: Good Bad

Are you use water after boiling: Yes No

Are you use home toilet or open toilet: Home toilet Open toilet

Where you put waste water and toilet waste: Pond (Gutter) Sewerage

What is the system of sewerage: Open Closed

What is the situation of cleanliness at your home: Good Bad

Are the household members wash hands with soap after using toilet: Yes No

If yes; with which soap: ______________________ (Specify name)

What is the frequency of illness in household members e.g. diarrhea or other diseases

i) Once in a week
ii) Once in a month
iii) Once in 3 months
iv) Once in 12 months

How many doctors are there in the village: ____________________________________

A.2. <u>Food consumption information for calorie intake calculations (Quantitative information to measure food security – calorie / protein intake method))</u>

A. Food Consumption related data, last 7 and 30 days

**Calorie chart for one household**					
**Sr. No.**	**English name**	**Urdu name**	**Quantity consumed (Kg) (7 days)**	**Quantity bought (Kg) (30 days)**	**Quantity Consumed (Kg) (30 days)**
**A.1) Bread related**					
1	Barley	Jou			
2	Rice	Chaval			
4	Wheat grain flour	Aata			
5	Wheat flour	Maida			
6	Wheat flour	Suji			
7	Biscuit	Biscuit			
**A.2) Garam masala**					
8	Garam masala	Masala			
**A.3) Fats and oil**					
9	Butter	Makhan			

10	Banaspati	Ghee			
11	Oil	Tail			
**A.4) Sugar, sweets and beverages**					
12	Sugar	Cheeni			
13	Gur				
**A.5) Legumes**					
14	Legumes (4)	Dalain			
**A.6) Vegetables**					
15	Onion	Piaz			
16	Potato	Aalu			
17	Vegetables	Sabzi			
**A.7) Fruits**					
18	Fruits	Phall			
**A.8) Meat**					
19	Beef	Ghosht			
20	Chicken	Ghosht			
21	Goat/Lamb	Ghosht			
22	Fish	Machli			
**A.9) Egg**					
23	Eggs (one piece)	Anda			
**A.10) Dairy products**					
24	Milk	Doodh			
25	Curd	Dehi			
26	Cheese	Paneer			
**A.11) Other food items which you consume commonly**					
27					
28					
29					
30					

31					
32					
33					

A.3. Household food security qualitative questions (during last 12 months)

Q.3.1. What do you consider as basic daily food requirement for your household?

____

____

____

Q.3.2. Considering basic daily food requirements as minimum food intake required for life: do you think that the basic daily food intake of your household has improved?

Yes No

If yes, to what extant?

a. Slightly better
b. Better
c. Much better

If no, then?

a. Same
b. Worse
c. Much worse

Q.3.3. Did you or other household members ever skip a meal because there was not enough money to buy food over last 12 months?

a. Once a week
b. Once a month
c. Once in 3 months
d. Once in 6 months
e. Never skipped the meal.
f. Don’t know

Q.3.4. Did you or other household members ever not eat for a whole day because of lack of money to buy food?

a. Once a week

b. Once a month
c. Once in 3 months
d. Once in 6 months
e. Never happened.
f. Don’t know

Q.3.5. Did you or other household members ever eat less than you or they would have needed to eat?

a. Once a week
b. Once a month
c. Once in 3 months
d. Once in 6 months
e. Never
f. Don’t know

Q.3.6. Sometimes people lose weight because of not having enough to eat. In the past 12 months did you lose weight due to not enough food?

a. Yes
b. No
c. Don’t know

Q.3.7. Did you ever cut the size of children’s meals due to not enough food available?

a. Once a week
b. Once a month
c. Once in 3 months
d. Once in 6 months
e. Never
f. Don’t know

Q.3.8. Did any of the children ever skip a meal due to not enough food?

a. Once a week
b. Once a month
c. Once in 3 months
d. Once in 6 months
e. Never

f. Don’t know

Q.3.9. Did any of the children ever not eat for a whole day because of lack of money to buy food?

a. Once a week
b. Once a month
c. Once in 3 months
d. Once in 6 months
e. Never
f. Don’t know

Q.3.10. “I worried whether our food would run out before we got money to buy more”. What was the frequency of this situation?

a. Once a week
b. Once a month
c. Once in 3 months
d. Once in 6 months
e. Never
f. Don’t know

Q.3.11. In your opinion what is a balanced meal?

____

____

____

Q.3.12. “We could not afford to eat balanced meals” what was the frequency?

a. Once a week
b. Once a month
c. Once in 3 months
d. Once in 6 months
e. Never
f. Don’t know

Q.3.13. “We could not feed the children a balanced diet due to lack of money” what was the frequency?

a. Once a week

b. Once a month
c. Once in 3 months
d. Once in 6 months
e. Never
f. Don't know

Q.3.14. How you dealt with such situation (food shortage) over last 12 months? (Tick all that apply)

a. Borrowing from friends, neighbors, relatives, etc.
b. Stick to simple food (e.g. dal roti or roti piaz)
c. Reduce expenditures on health
d. Reduce expenditures on education
e. Adults skip meals once a day
f. Selling assets (e.g. cycle, motorcycle, bull, farm equipment, etc)
g. Others (Please specify)

Q.3.15. Due to heavy flooding in the country general price level is expected to increase with expected shortage of food items, how you will deal with such situation, any planning?

Yes No

If yes, please explain

Q.3.16. Do you smoke?

Yes No

If yes

a. Less than half **a pack** per day
b. Half a pack daily
c. One pack daily
d. Two packs daily
e. Three packs daily

f. Four packs daily

'a pack' = 20 cigarettes

Q.3.17. Which brand do you normally smoke? ------------------------- (Please Specify)

**In the past 2 years, what difficulties have negatively impacted your household's ability to meet your food needs?**		
**Shock**	**Tick all the difficulties identified by the household**	**Rank the top 3 difficulties**
Loss of employment/ reduced salary		
Sickness/ health expenditures		
Death of household head		
High food prices		
Debt to reimburse		
Insecurity/ thefts		
Crop disease		
Animal disease / death		
Lack of irrigation water		
Bad climate(poor crop yield/harvest)		
Others (Please specify)		

A.4. Income and expenditure data.

A.4.1 Source of Income

<table>
<tr><td>Number of Earners</td><td></td><td><u>Amount (Rs.)</u></td></tr>
<tr><td rowspan="2">Farm Income (over <u>last 12 months</u>)</td><td>From crops</td><td></td></tr>
<tr><td>From Livestock</td><td></td></tr>
<tr><td rowspan="11">Off-farm Income</td><td colspan="2">Dehari dar</td></tr>
<tr><td>Number of HH Members</td><td></td></tr>
<tr><td>Wage rate (Rs. daily)</td><td></td></tr>
<tr><td>Average worked days per week</td><td></td></tr>
<tr><td colspan="2">Job Holders (Govt.)</td></tr>
<tr><td>Number of HH members</td><td></td></tr>
<tr><td>Wages (monthly)</td><td></td></tr>
<tr><td colspan="2">Job Holders (Private)</td></tr>
<tr><td>Number of HH members</td><td></td></tr>
<tr><td>Wages (monthly)</td><td></td></tr>
<tr><td></td><td></td></tr>
<tr><td colspan="3">Do you make a pool of incomes of all earning members? Yes No
If no, do you take share from each earning member to make a pool? Yes No
How much share? _______________</td></tr>
</table>

A.4.2 Household Expenditures

<table>
<tr><td colspan="2">Expenditures on (Rs.)</td><td>Monthly</td></tr>
<tr><td colspan="2">Food</td><td></td></tr>
<tr><td rowspan="2">Health</td><td>Doctor Fee</td><td></td></tr>
<tr><td>Medicine</td><td></td></tr>
<tr><td colspan="2">Clothing</td><td></td></tr>
<tr><td colspan="2">Education</td><td></td></tr>
<tr><td colspan="2">Weddings (Son, Daughter)</td><td></td></tr>
<tr><td colspan="2">Funerals</td><td></td></tr>
</table>

Taxes				
Weddings of relatives				
Expenditures on legal matters like courts, police station etc.				
Other major exp.				
Proportion of income spent on (%)	**Food**	**Health**	**Clothing**	**Education**

***A.4.3. Farm Production, Expenditure and Income Data**

∗Q.A.4.3.1. Which crops have you grown in last 12 months? (Tick all that apply)

a. Wheat
b. Barley
c. Rice
d. Cotton
e. Sugarcane
f. Maize
g. Vegetables

∗Q.A.4.3.2. Please mention the fertilizers you applied for each of these crops

**Crops**	**Urea** (Number of bags)	**DAP** (Number of bags)	**FYM** Number of Trolleys	**Others** (Number of bags)
**Price**				
Wheat				
Barley				
Rice				
Cotton				
Sugarcane				
Maize				
Vegetables				

∗**Q.A.4.3.**3. Please mention the seed rates you used for each of these crops

**Crops**	**Area Sown (acres)**	**Seed Used Kg/acre**	**Prices (Rs.)**
Wheat			
Barley			
Rice			
Cotton			
Sugarcane			
Maize			
Vegetables			

∗ Q.A.4.3.4. Please mention the plant protection treatments you applied for each of these crops

**Crops**	**No. of treatments / acre**	**Price / treatment / acre (Rs.)**	**Total area treated**
Wheat			
Barley			
Rice			
Cotton			
Sugarcane			
Maize			
Vegetables			

∗Q.A.4.3.5. Please mention the expenditures on irrigation for these crops

**Crops**	**Tube well**		**Canal Water (Aabyaana)** (Rs.)
	If Purchased = Cost (Rs.)	If owned at what price would you sell (Rs.)	
Wheat			
Barley			

Rice			
Cotton			
Sugarcane			
Maize			
Vegetables			

∗ Q.A.4.3.6. Please mention the expenditures on Labor/family labor

Wage = In cash _______________ Rs. per person per day

In Kind _______________ Kg. per person per day

Land Rent = (if applicable) ------------------------- (for a year) for ___________ acres

**Labor used for**	**Number of persons**	**Number of days**	**If different rates for sugarcane or other crops**
Sowing			
Weeding			
Harvesting			
Spray			

∗Q.A.4.3.7. Please mention the expenditures on Marketing of your produce

**Crops**	**Quantity (mds) marketed**	**Transportation Costs** (Rs.) to the nearest market	**Market charges** (Rs.) (fee, taxes, etc)
Wheat			
Barley			
Rice			
Cotton			
Sugarcane			
Maize			
Vegetables			
Other			

∗ Q.A.4.3.8. Please mention the expenditures on livestock

**Livestock**	**Numbers of animals (at the time of interview)**	**Feed (Rs. Per month)**	**Medicine (Rs. Per month)**	**Other expenditures (Rs.) (tools, fence replacements, etc.)**
Goats				
Sheep				
Buffalo				
Cow				
Oxen				
Chickens				
Horses/ donkeys				
Other				

Q.A.4.3.9. Please provide information about any loan taken during <u>last 12 months</u>

Loan Taken Yes No

Source: Banks Non-Bank (which one please mention -------)

Purpose: Production Personal Development

Interest rate ---------------------------

∗Q.A.4.3.10. Please mention the production per acre and sale price of your farm produce during <u>last 12 months</u>

**Crops**	**Production per acre** (Mds)	**Price (sold) per unit** (Rs/Md)
Wheat		
Barley		
Rice		
Cotton		
Sugarcane		
Maize		
Vegetables		

Others		

* Q.A.4.3.11. Please mention; production, consumption and sold livestock during last 12 months

Livestock Income	Production (Kg for Milk and Numbers for Meat and dozens for eggs)	Home Consumption (Kg for Milk and Numbers for Meat and dozens for eggs)	sold in market	
		Quantity	Quantity	Price (Rs.)
**Milk**				
Goat/ Sheep				
Cow				
Buffalo				
**Meat**				
Goat				
Sheep				
Buffalo				
Cow				
Oxen				
Chickens Meat				
Eggs (Dozens)				
Others				

(*) means 'skip if landless'

A.5. <u>Information about accessibility to input and output markets</u>

Q.5.1. Please mention the distance to nearest markets from your home/farm etc.

No.	Distance to nearest	Distance in Kilometres (Km)	Transportation Cost (1 time) in PKR	Transportation Cost (Last 12 months) in PKR

1	Tarred Road			
2	Seed and Fertilizer Market			
3	Pesticides Market			
4	Output Market (Mandi) your you sell your farm produce			
5	PASSCO Centre			
6	Utility Store			
7	Shop from where you purchased food stuff for home use daily			

Q.5.2. Do you have easy access and satisfied with the extension services of Punjab Agricultural Department?

Yes No

If Yes: to what extent

i) Dissatisfied

ii) Less Satisfied

iii) Satisfied

If No; what are the reasons

i) ………………………………………………………………………………………

ii) ………………………………………………………………………………………

iii) ………………………………………………………………………………………

Q.5.3. Do you have credit access?

Yes No

If No; what are the reasons

i) ………………………………………………………………………………………

ii) ………………………………………………………………………………………

iii) ………………………………………………………………………………………

Q.5.4. Do you have electricity access at your farm?

Yes No

Q.5.5. Do you think the extension workers have influence on your sales decisions?

Positive Negative

Q.5.6. Do you think cooperative societies have influence on your sales decisions?

Positive Negative

Q.5.7. Do you have access to market information?

Yes No

If Yes; to what extent

i) Negligible

ii) Very less

iii) less

iv) Enough

v) Full

If No; then what are the reasons

i) ………………………………………………………………………………………

ii) ………………………………………………………………………………………

iii) ………………………………………………………………………………………
