# Supplementary material for: Status and determinants of small farming households' food security and role of market access in enhancing food security in rural Pakistan
Source: PLoS One. 2017 Oct 27;12(10):e0185466. doi: 10.1371/journal.pone.0185466 (PMC5659641; doi:10.1371/journal.pone.0185466)
Supplement: S2 Table — (PDF) [file pone.0185466.s002.pdf]

S2 Table

Table. Dietary consumption units according to age and sex

Category	Consumption Unit
Adult male (Sedentary worker)	1.0
Adult male (Moderate worker)	1.2
Adult male (Heavy worker)	1.6
Adult female (Sedentary)	0.8
Adult female (Moderate)	0.9
Adult female (Heavy)	1.2
Adolescent (12–21 years)	1.0
Children (9–12 years)	0.8
Children (7–9 years)	0.7
Children (5–7 years)	0.6
Children (3–5 years)	0.5
Children (1–3 years)	0.4

Note: One consumption unit represents calorie consumption of an average adult man, weighing 60 kg, doing sedentary type of work, Source: [43]
