# Supplementary material for: Status and determinants of small farming households' food security and role of market access in enhancing food security in rural Pakistan
Source: PLoS One. 2017 Oct 27;12(10):e0185466. doi: 10.1371/journal.pone.0185466 (PMC5659641; doi:10.1371/journal.pone.0185466)
Supplement: S3 Table — (PDF) [file pone.0185466.s003.pdf]

S3Table

FOOD COMPOSITION TABLE FOR PAKISTAN (REVISED 2001)

Amount in 100g of edible portion

No	Name of Food	kcal	No	Name of Food	Kcal
a)	**Cereal and Cereal Products**		41	Lichi	62
1	Corn Whole grain flour	276	42	Mango Ripe	64
2	Rice Polished Fried	268	43	Melon Water	23
3	Vermicelli	345	44	Mandarin	44
4	Wheat Whole grain flour	357	45	Orange Sweet	43
5	Wheat flour Granular	370	46	Peach	47
6	Wheat Bread	369	47	Pomegranate	66
7	Wheat Bread	259	48	Zizyphus	79
8	Wheat Bread	364	G)	**Dairy Products**	
9	Wheat Bread	293	49	Butter Milk	31
10	Wheat Bread	263	50	Curd	52
11	Wheat Flour	440	51	Cream	361
B)	**Legumes**		52	Milk Buffalo Fluid Whole	105
12	Broad Bean Cooked	175	53	Milk Cow Fluid Whole	66
13	Chickpea Cooked	187	54	Milk Goat Fluid Whole	70
14	Lentil Cooked	178	55	Yogurt	71
15	Mung Bean Cooked	120	56	Ice-cream	148
16	Mash Cooked	158	H)	**Meat & Products**	
C)	**Vegetables**		57	Beef	244
17	Bath Sponge	18	58	Buffalo Meat	123
18	Bottle Gourd	15	59	Chicken Meat	187
19	Bringal	26	60	Goat Meat	164
20	Cauliflower	27	61	Sheep Meat	175
21	Cocumber	16	I)	Eggs	
22	Lady Finger	35	62	Chiken Egg White	400

23	Spinach	27	63	Duck Egg White (Raw)	895
24	Tinda	23	J)	**Fats & Oils**	
D)	**Roots & Tubers**		64	Butter	721
25	Carrots	37	65	Ghee	874
26	Onion	44	66	Ghee (Buffalo)	900
27	Potato	83	67	Lard (Raw)	899
28	Reddish	23	68	Dalda (Hydrogenated Oil)	892
29	Turnip	26	69	Corn Oil	900
E)	**Spices & Condiments**		70	Soybean	887
30	Cumin Seed	336	K)	**Sugar, Sweets & Beverages**	
31	Liquorice Root	212	71	Sugar	380
32	Clove	304	72	Gur	310
33	Turmeric	365	73	Honey	310
34	Pepper Black	268	74	Barfi	384
F)	**Fruits**		75	Jaleebe	395
35	Apple	57	76	Koa (Whole Buffalo Milk)	401
36	Banana Ripe	96	77	Halwa Sohen	481
37	Dates Dried	293	78	Carbonated Beverages Pepsi, Coke, etc.	39
38	Dates Fresh	131	79	Lemon Juice	43
39	Guava Whole	73	80	Mango Juice	74
40	Lemon	30			

Source: AIOU 2001
